# Supplementary material for: Pathogenic impact of transcript isoform switching in 1,209 cancer samples covering 27 cancer types using an isoform-specific interaction network
Source: Sci Rep. 2020 Sep 2;10:14453. doi: 10.1038/s41598-020-71221-5 (PMC7468103; doi:10.1038/s41598-020-71221-5)
Supplement: Supplementary file 1 — Supplementary Information. [file 41598_2020_71221_MOESM1_ESM.docx]

Supplementary materials

**Pathogenic impact of transcript isoform switching in 1209 cancer samples covering 27 cancer types using an isoform-specific interaction network**

Abdullah Kahraman^1,2,3^, Tülay Karakulak^1,2,3^, Damian Szklarczyk^1,3^, Christian von Mering^1,3,*^

^1^ University of Zurich, Institute of Molecular Life Sciences (Zurich, Switzerland)

^2^ University Hospital Zurich, Department of Pathology and Molecular Pathology, Molecular Tumor Profiling lab (Zurich, Switzerland)

^3^ Swiss Institute of Bioinformatics

^*^ Correspondence to [mering@imls.uzh.ch]

# Supplementary Results

Most dominant transcript switches as diagnostic biomarkers

In prostate cancer, we identified the transcript ENST00000361518 of Zink Finger Protein 511 (ZNF511) as a cMDT in all 19 PCAWG samples, while the 10 AA longer alternative transcript ENST00000359035 was found as MDT in normal prostate GTEx samples (see Figure 3C). Both protein isoforms differ only in their C-terminal region. Interestingly, ZNF511 was already previously found to be differently expressed in prostate cancer, where it was part of NF-kB-activated cancer recurrence predictors 48. Our analysis confirms their findings and extents it further with the identification of ENST00000361518 as a Prost-AdenoCA specific transcript of ZNF511. Another highly interesting case was the transcript ENST00000538098 from the gene WDR74, which was the cMDT in 100% of all Panc-AdenoCA cases (see Table 1). While the transcript had no expression in almost all normal GTEx samples of the pancreas, the median expression in PCAWG was 80463.07 TPM, which might indicate an important role of WDR74 in Panc-AdenoCA.

## Cancer-specific most dominant transcripts disrupting protein-protein interactions

The mitochondrial NADH dehydrogenase (ubiquinone) alpha subcomplex 9 (NDUFA9) was also mainly expressed via an alternative promoter in 93% Uterus-AdenoCA samples and 35% of ColoRect-AdenoCA samples. As a result, the cancer-specific transcript ENST00000540688 was 136 AA long and lacked the first 235 AA of the longer GTEx-specific transcript ENST00000266544. Not only was ENST00000540688 shorter, but the first 57 AA were also encoded by an alternative exon, making the N-terminus of the cancer-specific transcript distinct from the GTEx MDT. As a result, most of the canonical protein sequence including the binding site sequence for NDUFS7 was missing in the cancer-specific transcript of NDUFA9 (see Figure 4D). Thus, in the 66 PCAWG cancer samples, NDUFA9 is not able to interact with NDUFS7, which will destabilize the structure and function of the Respiratory complex I, impacting the electron transfer from NADH to ubiquinone. Various germline mutations in NDUFA9 are known to cause severe neurological disorders ^1^. In breast cancer cell-lines, dysregulation of the NAD+/NADH balance was found to correlate with enhanced cancer progression ^2^. Thus, we postulate that the short cancer-specific NDUFA9 transcript causes mitochondrial respiratory defects, which could promote aerobic glycolysis in the effected cancer cells leading to cancer progression ^3^.

## Pathogenic disruptions of protein interactions due to alternative splicing

In the case of CDK4, the 111 amino acid short cMDT (ENST00000312990), which lacks the entire C-lobe of the kinase domain from the GTEx-specific transcript (ENST00000257904) was expressed in 36% of Uterus-AdenoCA and 14% in Eso-AdenoCA. The loss of the C-lobe disrupted the kinase activity of CDK4. In CDK4 knock-out mice, the loss of CDK4 function has mild effects on cell cycle progression, due to CDK6 compensating for CDK4 loss ^4^. However, in 11 of 24 samples, this compensation effect is most likely absent due to mutated CDK6, which hints towards a strong functional impact of CDK4 cMDT in these tumors. CDK4 lies in the top 11% of densest network regions in STRING.

## Discovering novel pathogenic genes via cancer-specific most dominant transcripts

The Charged multivesicular body protein 7 (CHMP7) was found to have a cMDT (ENST00000517325) in 50% of Uterus-AdenoCA, 17% of Breast-LobularCA and 13% of Breast-AdenoCA. It is part of the densest 22% of network regions in STRING. According to the Ensembl database also this cMDT is predicted to undergo nonsense-mediated decay. As a result, the interaction between CHMP7 and other CHMP family members (2A, 2B, 3, 4A, 4B, 4C, 5, 6) and the Vacuolar protein sorting-associated homolog protein VTA1 is deleted. CHMP7 is known to play an important role in repairing envelope raptures after cancer cell migration ^5^. Lacking functional CHMP7 proteins in cancer cells and thus a fully functional nuclear envelope can induce extensive double-strand breaks and damage to nuclear DNA ^6^. Thus, the NMD driven loss of CHMP7 could play an important role in the cancer hallmark describing genome instability and mutations ^7^.

Non-coding mutations associated with cancer-specific most dominant transcripts

Transcripts in Lymph-BNHL whose over-expression significantly correlated with mutations in cis were those of MYC (see Figure S2B and Figure S3) with mutations in 5’UTR, promoter, splice-site in particular exonic mutations (ENST00000377970 (FDR corrected Wilcox test = 4.0e-05), ENST00000524013 (FDR corrected Wilcox test = 5.3e-05)) and those of Serum/glucocorticoid-regulated kinase 1 (SGK1) with various mutations in the promotor, UTR regions, splice site and coding sequence (ENST00000460769 (FDR corrected Wilcox test = 0.005), ENST00000367858 (FDR corrected Wilcox test = 0.008)) (Figure S2).

Over the 55 Panc-AdenoCA samples, we found a significant correlation between expressions of the CDKN2A transcripts ENST00000479692 and ENST00000497750 and 22 mutations covering the entire coding sequence and a single splice site mutation in the second last exon (ENST00000479692 (FDR corrected Wilcox test = 0.004, ENST00000497750 (FDR corrected Wilcox test = 0.008)) (see Figure S2C and Figure S3).

Additional correlations between expression and mutations were identified for the canonical transcript of TERT (ENST00000310581) whose expression significantly correlated with mutations in the promoter region of 11/47 Thy-AdenoCA samples (FDR corrected Wilcox test = 5.5e-06) (see Figure S2D). The expression of the transcripts ENST00000547379 and ENST00000367714 from the Monocarboxylate transporter gene SLC16A7 and Sodium/hydrogen exchanger gene SLC9C2, respectively, were significantly correlated with various mutations in 10 and 8 ColoRect-AdenoCA samples, respectively (ENST00000547379 (FDR corrected Wilcox test = 0.002), ENST00000367714 (FDR corrected Wilcox test = 0.005)) (see Figure S2E and S2F). However, the median expression of these transcripts and the TERT transcript was generally below 2 TPM, which was the threshold for transcripts to be included in our study. Thus, these transcripts were not considered for cMDT analysis.

# Supplementary Figures

*Figure S1: Number of short nucleotide variances, i.e. single/multi nucleotide variances and indels, per cancer type in log-scale. The top axis represents the sum of all mutations per cancer type. Red lines show the median number of mutations per cancer type.*

 *Figure S2: Integrating PCAWG Whole Genome Sequencing data with Most Dominant Transcript (MDT) information. Shown are transcripts whose expression most significantly correlated with mutations in the gene structure. q-values are FDR corrected p-values from Wilcox-Rank sum tests between PCAWG expression values from mutated samples vs. non-mutated samples. Please see* ^8,9^ *for the color code. Upper-case N* is the number of samples used for correlation analysis, while *lower-case n* is the number of mutations identified in the mutated samples.

**

*Figure S3: Transcripts whose expression significantly correlated with mutations in their associated gene structure. These transcripts are part of multiple transcripts from the same gene that all show a significant correlation between expression and gene mutation (compare to Figure S2).*

**

*Figure S4: Distribution of number of cMDT across samples having mutations on genes with Spliceosomal Complex GO term. The samples with mutation in a protein of spliceosome complex demonstrates higher number of cMDT. The significance of number of cMDT differences in samples without mutation and with mutations was p-value 1.08e-06 (Wilcox-Rank sum test).*

# Supplementary tables

*Table S1: Mapping table between PCAWG code, PCAWG cancer type name and matched GTEx tissue cohort.*

| ***PCAWG code*** | ***Cancer type name*** | ***GTEx tissue*** |
| --- | --- | --- |
| *Biliary-AdenoCA* | *Biliary Adenocarcinoma* | *Liver* |
| *Bladder-TCC* | *Bladder Transitional Cell Carcinoma* | *Bladder* |
| *Bone-Leiomyo* | *Bone/Soft-tissue Leiomyosarcoma* | *Muscle* |
| *Breast-AdenoCA* | *Breast Adenocarcinoma* | *Breast* |
| *Breast-LobularCA* | *Breast Lobular Carcinoma* | *Breast* |
| *Cervix-AdenoCA* | *Cervix Adenocarcinoma* | *Cervix-Uteri* |
| *Cervix-SCC* | *Cervical Squamous Cell Carcinoma* | *Cervix-Uteri* |
| *CNS-GBM* | *CNS Glioblastoma* | *Brain* |
| *CNS-Oligo* | *CNS Oligodendroglioma* | *Brain* |
| *ColoRect-AdenoCA* | *Colon/Rectum Adenocarcinoma* | *Colon* |
| *Eso-AdenoCA* | *Esophagus Adenocarcinoma* | *Esophagus* |
| *Head-SCC* | *Head/Neck Squamous Cell Carcinoma* | *Salivary-Gland* |
| *Kidney-ChRCC* | *Kidney Renal Cell Carcinoma, Chromophobe type* | *Kidney* |
| *Kidney-RCC* | *Kidney Renal Cell Carcinoma, clear cell and papillary* | *Kidney* |
| *Liver-HCC* | *Liver Hepatocellular Carcinoma* | *Liver* |
| *Lung-AdenoCA* | *Lung Adenocarcinoma* | *Lung* |
| *Lung-SCC* | *Lung Squamous Cell Carcinoma* | *Lung* |
| *Lymph-BNHL* | *Lymphoid Mature B-cell Lymphoma* | *Blood* |
| *Lymph-CLL* | *Lymphoid Chronic Lymphocytic Leukaemia* | *Blood* |
| *Lymph-NOS* | *Lymphoma - Not Otherwise Specified* | *Blood* |
| *Ovary-AdenoCA* | *Ovarian Adenocarcinoma* | *Ovary* |
| *Panc-AdenoCA* | *Pancreas Adenocarcinoma* | *Pancreas* |
| *Prost-AdenoCA* | *Prostate Adenocarcinoma* | *Prostate* |
| *Skin-Melanoma* | *Skin Melanoma* | *Skin* |
| *Stomach-AdenoCA* | *Stomach Adenocarcinoma* | *Stomach* |
| *Thy-AdenoCA* | *Thyroid Adenocarcinoma* | *Thyroid* |
| *Uterus-AdenoCA* | *Uterus Adenocarcinoma* | *Uterus* |

*Table S2: (see TableS2.csv in Supplementary Materials). Isoform-specific interaction network with information on which interactions are lost and which remain for each alternatively spliced isoform/transcript. Please read the comment in the file’s header for more information on the file format.*

*Table S3: (see TableS3.csv in Supplementary Materials) List of all detected cancer-specific Most Dominant Transcripts (cMDT) and the protein interactions they disrupt with a rich set of functional annotations.*

*Table S4: (see TableS4.csv in Supplementary Materials) Most dominant transcripts found in the PCAWG dataset. The Ensembl Gene ID, as well as the gene name, are listed. The number of samples in which the cMDT was observed in the cancer type is given in the Frequency column. The total number of samples per cancer type is listed in the 6th column, followed by the percentage of samples expressing the transcript as cMDT.*

*Table S5: (see TableS5.csv in Supplementary Materials) Disrupted protein interactions due to cancer-specific Most Dominant Transcripts (cMDT) in the PCAWG dataset.*

*Table S6: (see TableS6.csv in Supplementary Materials) Most significant Gene Ontology biological processes found to be enriched in cancer-specific Most Dominant Transcripts (cMDT) disrupting protein interactions. Enrichment analysis with FDR corrected p-values were computed on the STRING interaction network using the STRINGdb R-package*

^10^*.*

*Table S7: Spearman correlation coefficients R and p-value between the number of short variants and number of cancer-specific Most Dominant Transcripts (cMDT) for all cancer types. The scatter plot of all points is shown in Figure S1. A correlation coefficient for SalivaryGland/Head-SCC could not be computed, as it lacked any detectable cMDT.*

| ***Cancer types*** | ***R-correlation*** | ***p-value*** |
| --- | --- | --- |
| *CervixUteri/Cervix-AdenoCA* | *-1.00* | *1* |
| *Colon/ColoRect-AdenoCA* | *-0.23* | *0.109* |
| *Liver/Biliary-AdenoCA* | *-0.10* | *0.683* |
| *Bladder/Bladder-TCC* | *-0.09* | *0.673* |
| *Kidney/Kidney-RCC* | *-0.07* | *0.486* |
| *Uterus/Uterus-AdenoCA* | *-0.06* | *0.716* |
| *Liver/Liver-HCC* | *-0.02* | *0.861* |
| *Thyroid/Thy-AdenoCA* | *-0.02* | *0.914* |
| *Esophagus/Eso-AdenoCA* | *0.00* | *1* |
| *Pancreas/Panc-AdenoCA* | *0.01* | *0.933* |
| *Brain/CNS-GBM* | *0.02* | *0.923* |
| *Blood/Lymph-BNHL* | *0.04* | *0.708* |
| *Muscle/Bone-Leiomyo* | *0.05* | *0.784* |
| *Stomach/Stomach-AdenoCA* | *0.10* | *0.62* |
| *Blood/Lymph-CLL* | *0.11* | *0.389* |
| *Kidney/Kidney-ChRCC* | *0.13* | *0.422* |
| *CervixUteri/Cervix-SCC* | *0.15* | *0.545* |
| *Lung/Lung-AdenoCA* | *0.16* | *0.34* |
| *Skin/Skin-Melanoma* | *0.18* | *0.295* |
| *Lung/Lung-SCC* | *0.19* | *0.212* |
| *Breast/Breast-AdenoCA* | *0.25* | *0.0193* |
| *Brain/CNS-Oligo* | *0.28* | *0.256* |
| *Ovary/Ovary-AdenoCA* | *0.28* | *0.00288* |
| *Prostate/Prost-AdenoCA* | *0.28* | *0.247* |
| *Breast/Breast-LobularCA* | *0.49* | *0.356* |
| *Blood/Lymph-NOS* | *1.00* | *1* |
| *SalivaryGland/Head-SCC* | *NA* | *NA* |

*Table S8: Transcripts showing expression correlation with mutation in cis. q-values are FDR-corrected p-values, which have been computed using the non-parametric Wilcox-rank sum test on the expression values in mutated and wildtype samples.*

| ***Cancer types*** | ***ENSEMBL transcript identifier*** | ***Gene name*** | ***Number of mutated samples*** | ***Number of wildtype samples*** | ***q-value*** |
| --- | --- | --- | --- | --- | --- |
| *Breast-AdenoCA* | *ENST00000512332* | *CPNE4* | *5* | *80* | *5.2e-04* |
| *ColoRect-AdenoCA* | *ENST00000547379* | *SLC16A7* | *10* | *41* | *1.9e-03* |
| *ColoRect-AdenoCA* | *ENST00000367714* | *SLC9C2* | *8* | *43* | *5.1e-03* |
| *Kidney-RCC* | *ENST00000413894* | *MID1* | *5* | *112* | *2.9e-03* |
| *Liver-HCC* | *ENST00000543084* | *TRIP12* | *8* | *92* | *2.0e-03* |
| *Liver-HCC* | *ENST00000524020* | *HACE1* | *5* | *95* | *2.6e-03* |
| *Lung-SCC* | *ENST00000305165* | *REG3A* | *8* | *39* | *8.3e-03* |
| *Lung-SCC* | *ENST00000425756* | *NCK2* | *5* | *42* | *2.1e-03* |
| *Lymph-BNHL* | *ENST00000398117* | *BCL2* | *44* | *59* | *3.6e-05* |
| *Lymph-BNHL* | *ENST00000333681* | *BCL2* | *44* | *59* | *2.4e-08* |
| *Lymph-BNHL* | *ENST00000589955* | *BCL2* | *44* | *59* | *1.6e-07* |
| *Lymph-BNHL* | *ENST00000377970* | *MYC* | *29* | *74* | *4.0e-05* |
| *Lymph-BNHL* | *ENST00000524013* | *MYC* | *29* | *74* | *5.3e-05* |
| *Lymph-BNHL* | *ENST00000367858* | *SGK1* | *19* | *84* | *7.5e-03* |
| *Lymph-BNHL* | *ENST00000460769* | *SGK1* | *19* | *84* | *4.6e-03* |
| *Lymph-BNHL* | *ENST00000394797* | *CHRDL1* | *5* | *98* | *2.3e-04* |
| *Panc-AdenoCA* | *ENST00000479692* | *CDKN2A* | *20* | *55* | *3.6e-03* |
| *Panc-AdenoCA* | *ENST00000497750* | *CDKN2A* | *20* | *55* | *7.8e-03* |
| *Panc-AdenoCA* | *ENST00000367170* | *NFASC* | *7* | *68* | *2.2e-03* |
| *Thy-AdenoCA* | *ENST00000310581* | *TERT* | *11* | *36* | *5.5e-06* |

Table S9: (see TableS9.csv in Supplementary Materials) Cancer-type-specific most dominant transcripts that are never found as most dominant transcripts in GTEx normal samples and are unique to a cancer type.

# References

1. Baertling, F. *et al.* NDUFA9 point mutations cause a variable mitochondrial complex I assembly defect. *Clin. Genet.* **93,** 111–118 (2018).

2. Santidrian, A. F. *et al.* Mitochondrial complex I activity and NAD+/NADH balance regulate breast cancer progression. *J. Clin. Invest.* **123,** 1068–1081 (2013).

3. Srinivasan, S., Guha, M. & Avadhani, N. G. Mitochondrial respiratory defects promote the Warburg effect and cancer progression. *Mol Cell Oncol* **3,** e1085120 (2016).

4. Berthet, C. & Kaldis, P. Cell-specific responses to loss of cyclin-dependent kinases. *Oncogene* **26,** 4469–4477 (2007).

5. Denais, C. M. *et al.* Nuclear envelope rupture and repair during cancer cell migration. *Science* **352,** 353–358 (2016).

6. Willan, J. *et al.* ESCRT-III is necessary for the integrity of the nuclear envelope in micronuclei but is aberrant at ruptured micronuclear envelopes generating damage. *Oncogenesis* **8,** 29 (2019).

7. Hanahan, D. & Weinberg, R. A. Hallmarks of Cancer: The Next Generation. *Cell* **144,** 646–674 (2011).

8. Gonzàlez-Porta, M., Frankish, A., Rung, J., Harrow, J. & Brazma, A. Transcriptome analysis of human tissues and cell lines reveals one dominant transcript per gene. *Genome Biol* **14,** R70 (2013).

9. Ezkurdia, I. *et al.* Most highly expressed protein-coding genes have a single dominant isoform. *J Proteome Res* **14,** 1880–1887 (2015).

10. Wang, H. *et al.* Identification of an exon 4-deletion variant of epidermal growth factor receptor with increased metastasis-promoting capacity. *Neoplasia* **13,** 461–471 (2011).
